# Molluscicidal and parasiticidal activities of *Eryngium triquetrum* essential oil on *Schistosoma mansoni* and its intermediate snail host *Biomphalaria glabrata*, a double impact

**DOI:** 10.1186/s13071-020-04367-w

**Published:** 2020-09-23

**Authors:** Ronaldo de Carvalho Augusto, Nadjiya Merad, Anne Rognon, Benjamin Gourbal, Cédric Bertrand, Nassim Djabou, David Duval

**Affiliations:** 1 University Perpignan Via Domitia, IHPE, UMR 5244, CNRS, IFREMER, Perpignan, France; 2 University Montpellier, IHPE, UMR 5244, CNRS, IFREMER, Montpellier, France; 3 Faculté des Sciences, Département de Chimie, Université de Tlemcen, Laboratoire COSNA, Tlemcen, Algeria; 4 EPHE-UPVD-CNRS, USR 3278 CRIOBE, Université de Perpignan, Perpignan, France; 5 Laboratoire d’Excellence «CORAIL», Université de Perpignan, Perpignan, France; 6 S.A.S. AkiNaO, Perpignan, France

**Keywords:** *Biomphalaria glabrata*, *Eryngium triquetrum*, Molluscicide, Oil, Parasiticide, *Schistosoma mansoni*

## Abstract

**Background:**

Freshwater snails are the intermediate hosts of a large variety of trematode flukes such as *Schistosoma mansoni* responsible for one of the most important parasitic diseases caused by helminths, affecting 67 million people worldwide. Recently, the WHO Global Vector Control Response 2017–2030 (GVCR) programme reinforced its message for safer molluscicides as part of required strategies to strengthen vector control worldwide. Here, we present the essential oil from *Eryngium triquetrum* as a powerful product with molluscicide and parasiticide effect against *S. mansoni* and the snail intermediate host *Biomphalaria glabrata.*

**Methods:**

In the present study, we describe using several experimental approaches, the chemical composition of *E. triquetrum* essential oil extract and its biological effects against the snail *B. glabrata* and its parasite *S. mansoni*. Vector and the free-swimming larval stages of the parasite were exposed to different oil concentrations to determine the lethal concentration required to produce a mortality of 50% (LC_50_) and 90% (LC_90_). In addition, toxic activity of this essential oil was analyzed against embryos of *B. glabrata* snails by monitoring egg hatching and snail development. Also, short-time exposure to sublethal molluscicide concentrations on *S. mansoni* miracidia was performed to test a potential effect on parasite infectivity on snails. Mortality of miracidia and cercariae of *S. mansoni* is complete for 5, 1 and 0.5 ppm of oil extract after 1 and 4 h exposure.

**Results:**

The major chemical component found in *E. triquetrum* oil determined by GC-FID and GC/MS analyses is an aliphatic polyacetylene molecule, the falcarinol with 86.9–93.1% of the total composition. The LC_50_ and LC_90_ values for uninfected snails were 0.61 and 1.02 ppm respectively for 24 h exposure. At 0.5 ppm, the essential oil was two times more toxic to parasitized snails with a mortality rate of 88.8 ± 4.8%. Moderate embryonic lethal effects were observed at the concentration of 1 ppm. Severe surface damage in miracidia was observed with a general loss of cilia that probably cause their immobility. Miracidia exposed 30 min to low concentration of plant extract (0.1 ppm) were less infective with 3.3% of prevalence compare to untreated with a prevalence of 44%.

**Conclusions:**

Essential oil extracted from *E. triquetrum* and falcarinol must be considered as a promising product for the development of new interventions for schistosomiasis control and could proceed to be tested on Phase II according to the WHO requirements. 
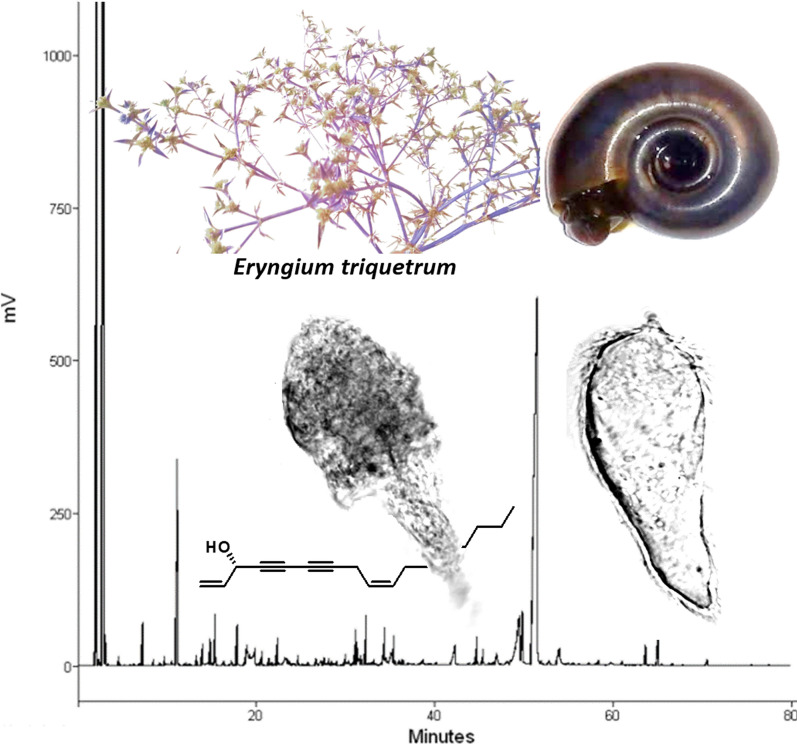

## Background

Water-borne infectious diseases, mainly those involving digenetic parasites, are mediated by both ecological and socioeconomic processes, and constitute an important public health problem in developing countries [1]. Schistosomiasis is one of the most common parasitic diseases worldwide [2]. It is particularly prevalent among people living in rural or deprived urban or peri-urban settings with limited access to clean water and with inadequate sanitation provision [3, 4]. Digenetic schistosomes have complex life-cycles entailing both mammalian hosts, including humans, and freshwater snail hosts. During their development, schistosomes have to deal with organic and inorganic compounds once in freshwater and then must face a multitude of molecules once inside both hosts [5]. For the control of schistosomiasis, the World Health Organization (WHO) recommends the mass administration of the chemotherapeutic praziquantel (PZQ), but also health education, technical cooperation between health institutions and research to verify the elimination of infection, and control of snail populations [2, 6].

The complexity of the parasite life-cycle gives rise to a complex list of requirements to obtain a successful control programme. Traditional models based exclusively on the use of schistosomicidal drugs have proven to significantly reduce morbidity in infected individuals, but the prevalence in the field remains high after decades of treatment. Indeed, continuous mass drug administration (MDA) campaigns in Africa and Brazil with PZQ, were not successful to eradicate the disease and a high prevalence and territorial expansion of infection areas are still detected worldwide [7]. Besides this, control strategies based only on one trial are struggling and deserve special attention since cases of rapid adaptation of the parasites to various treatment or specific environmental cues have been documented [5, 7–10]. To integrate MDA approaches, the WHO recommends the use of the synthetic molluscicide niclosamide (Bayluscide^®^) as one of the strategies to fight schistosomiasis in the field [2]. However, niclosamide has high a production value and can induce several secondary effects, such as bioaccumulation and high toxicity in non-target animals [11]. Also, snails resistant to niclosamide appeared recently and thus finding new molluscicides becomes necessary [12]. It is currently expected that we will see the development of a new class of safer molluscicides, which should be less harmful to the non-target organisms and, if possible, will be more selective to snails infected with schistosomes [13]. Increasing efforts have been made to focus on identifying and characterizing plant-derived molluscicides as safer alternatives [14–19].

In this study, we have focused on an essential oil extracted from *Eryngium triquetrum*. This Apiaceae is an endemic species of northern Africa, distributed in all parts of Algeria; the green perennial plant becomes a bluish-purplish color when ripe. This species grows particularly well in rocky pastures. It was considered as a ruderal species, which the local people call ‘choukzerk’ [20]. No traditional use for this thorny plant has been reported by the Algerian population. However, species of the genus *Eryngium* are used as a folk remedy for the treatment of various inflammatory disorders, and as an emetic infusion, antidote for poisons, hypoglycemic, antitussive and diuretic agents [21–24]. Species of *Eryngium* have been subjects of many phytochemical studies. For example, hexanic extract of *E. campestre* showed anti-trypanosomal and leishmanial activities [23]. *Eryngium caeruleum* extract displayed antimicrobial activities [25]. The essential oil of *E. triquetrum* for which the chemical composition was reported exhibits a moderate antibacterial action against Gram-positive and Gram-negative bacteria [23].

In order to further analyze the biological activity of *E. triquetrum*, we characterize and investigate the toxic effects of its essential oil against *S. mansoni* free-swimming stages (miracidia and cercariae) and on adults and embryos of the snail *B. glabrata*. Our results suggest that this biological compound could be used as a biological control agent against *B. glabrata* snails and its parasite *S. mansoni*.

## Methods

### Plant material and essential oil extraction

The fresh stems of *E. triquetrum* were harvested from June 2017, from three localities of northwest Algeria (Samples ET01-ET03) as described in the Table 1.

**Table 1:** Sample location of *Eryngium triquetrum* essential oil from three distinct geographical localities in Algeria

	ET01	ET02	ET03
Locality	Bouhanak	Ain fezza	Mefrouch
Latitude	35°08′16.22″N	34°52′37.96″N	34°50′59.19″N
Longitude	1°25′20.67″W	1°14′07.62″W	1°17′46.06″W
Altitude (m)	313	853	1108

The essential oil extractions were performed using a Clevenger-type apparatus according to the method recommended in the European Pharmacopoeia [26]. A sample of 400–500 g of the fresh plant was subjected to hydrodistillation.

### Compound identification

The identification of individual components was based on: (i) the comparison of the retention indices (RIs) determined on nonpolar columns with those of authentic compounds or literature data [27]; (ii) computer matching of the mass spectra with commercial mass-spectral libraries; and (iii) the comparison of the mass spectra with those listed in our in-house library built of mass spectra of authentic compounds or literature data [28].

### Compound quantification

Quantification of the oil components was performed using the methodology reported by Rubiolo et al. [29] and adapted in our laboratory [30]. Briefly, the compound quantification was carried out using peak normalization, including FID response factors (RFs) relative to tridecane (0.7 g/100 g) used as internal standard, and expressed as normalized contents (% abundances).

### Gas chromatography

Essential oil samples were analyzed with Thermo Scientific Focus GC with Detector Flame Ionization (FID) using an SPB-1 column fused silica capillary (30 m × 0.25 mm × 0.25 μm) with helium as the carrier gas (flow rate 1.0 ml/min) and an injection volume of 1 μl (solution in cyclohexane); the initial pressure was 1.0 Pa. The oven temperature was programmed as follows: 60 °C for 3 min, 60–240 °C (3 °C min^−1^), 240–300 °C (10 °C min^−1^) and 300 °C for 10 min. The injector temperature was 235 °C, the detector temperature was 300 °C with injection in the ‘splitless’ mode. The analyses lasted 80 min. Samples were analyzed in triplicate. The retention indices (RIs) of the compounds were determined related to the retention times (RT) of a series of n-alkanes (C7-C40) using the Van den Dool-Kratz equation.

### Gas chromatography-mass spectrometry

Thermo Scientific DSQ™ II gas chromatography coupled with a mass spectrometry detector, was performed with the same conditions that were previously described in Gas Chromatography. MS data were collected and processed on an Xcalibur™ version 1.4.1. For mass spectrometry conditions, the electron ionization was 70 eV.

### Experimentals animals

*Biomphalaria glabrata* originating from Recife, Brazil (BgBRE2), was used for this study. These snails were maintained in rearing chambers at 26 °C, 12:12 h light:dark photoperiod and fed *ad libitum* with fresh lettuce. A Puerto Rican strain (NMRI) of *S. mansoni* was used in this study to determine the effects of molluscicide on the susceptibility of infected snails and host-parasite compatibility.

### Material collection and extraction of essential oil from *Eryngium triquetrum*

Control experiments were performed with 1% dimethyl sulphoxide (DMSO) diluted with distilled and sterile water. Each essential oil (ET01, ET02 and ET03) was dissolved in DMSO to obtain different solutions in the following concentrations: 1 g/ml, 200 mg/ml, 40 mg/ml, 10 mg/ml, 2 mg/l, 0.2 mg/ml and 0.02 mg/ml. Then, each stock solution was diluted in natural mineral water to prepare the different test solutions.

### Molluscicidal bioassays

Biological assays against the snail *B. glabrata* were carried out following Phase I of stages of evaluation of molluscicides methods recommended by the WHO [31]. Briefly, 12 snails with shell diameters of 6–8 mm were individually exposed to different concentrations of essential oil or DMSO used as control into 12-well tissue culture plate (about 5.5 ml). All snails were immersed during 24 h and then washed and transferred to acrylic tanks filled with 5 liters with well water. Snails were fed with fresh lettuce *ad labitum* during the observation period (48 h). Each snail was observed under binocular microscope to appreciate the heart movements. No difference in mortality was found between the different recovery periods, 24 or 48 h respectively. To determine the lethal concentration LC_50_, Probit analysis was used from the three independent experiments performed with the different extract of essential oil.

Concerning assays on infected snails, each snail was exposed to 5 miracidia from the NMRI strain of *S. mansoni*. The procedures of miracidial recovery and snail infection were as conducted and described by Mone et al. [32]. Snail infection status was assessed by direct observation of daughter sporoscyts through the shell of exposed snails. After 15 days of exposure to parasites, 20–30 infected snails were submitted to different doses of essential oil from *E. triquetrum* to compare their toxic sensitivity with non-infected snails. This experiment was repeated three times at different times by different experimenters.

### Miracidiacidal and cercariacidal properties of essential oil from *Eryngium triquetrum*

Miracidia from NMRI strain were hatched from eggs recovered from hamster livers after 50 days post-infection. Eggs were filtered and washed to obtain miracidia which were collected under illumination. About 300 freshly hatched miracidia were used to test the toxicity of the essential oil from *E. triquetrum.* Swimming behavior and mortality were investigated after 1 and 4 h of treatments. All observations were done using Zeiss LSM microscope and stereoscopic microscope.

Infected *B. glabrata* (BgBre2) were exposed to artificial light to induce cercarial emission. About 100 cercariae were collected and exposed to different concentrations of essential oil in duplicates. Immobile cercariae were considered as dead.

### Impact of the active component derived from *Eryngium triquetrum* on *S. mansoni*-*B. glabrata* compatibility

The freshly hatched miracidia were recovered and exposed to 0.5 or 0.1 ppm of essential oil for 30 min. Then, each miracidium was collected individually to be placed with a single snail in 5 ml of dechlorinated water. For each condition, 42 snails were exposed individually to 1 miracidium which was treated with essential oil or left untreated. Prevalence was determined for each condition 40 days post-infection as described above.

### Toxicity of *Eryngium triquetrum* essential oil on *B. glabrata* embryos

Toxicity of the essential oil was evaluated against egg masses exposed during 24 h at three concentrations (1, 0.5 and 0.1 ppm). Embryonic development of the snails in egg masses was followed for 20 days under a binocular microscope after egg hatching. Polystyrene sheets served as substrate for oviposition. Egg masses were collected gently to avoid damaging them and maintained in Petri dishes with Volvic water. Twenty-four hours after exposition, the egg masses were washed with Volvic water and placed in a cell culture plate (Sarstedt). No selection of egg masses has been done according to the stage of development. The presence of all embryonic stages (blastula, gastrula, trochophore, veliger and hippo stage) has been checked. This toxicity test was carried out twice on more than 25 egg masses per condition.

### Statistical analysis

In all biological assays, at least three independent experiments were conducted at different times. The results were expressed as the mean ± standard deviation and subjected to the Fischer’s exact test. The lethal dose concentrations (LC_90_ and LC_50_) for each essential oil were calculated using a program employing probit analysis [33].

## Results

### Chemical characterization of the essential oil extracted from *Eryngium triquetrum*

The sampling of plant material in the three locations of northwest Algeria has allowed the determination of the species with large availability. Hydrodistillation from the fresh aerial parts of the three Algerian samples yields respectively (w/w) 0.05%, 0.04% and 0.03% of essential oils. GC-FID and GC/MS analyses of the three samples and subsequent fractions obtained by column chromatography allowed the identification of 24 components, accounting for more than 95% of the total composition (Table 2). The essential oil contains mostly linear oxygenated compounds, dominated by falcarinol (86.9–93.1%), followed by octanal (1.0–1.8%) and nonanal (0.2–0.4%). Twenty-two components were identified by comparison of their retention indices and mass spectra with those in an in-house mass spectral library.

**Table 2:** Percentage of different fractions of *Eryngium triquetrum* essential oil from three distinct geographical localities in Algeria

No	Components^a^	RI_a_^b^	*Eryngium triquetrum*			
			ET01^c^	ET02^c^	ET03^c^	Identification
1	Heptane	703	0.2	0.1	0.1	RI, MS
2	Hexanal	774	0.2	0.2	0.2	RI, MS
3	Heptanal	877	tr	tr	tr	RI, MS
4	Octanal	979	1.8	1.0	1.0	RI, MS
5	(E)-2-octenal	1039	tr	tr	tr	RI, MS
6	1-octanol	1063	0.3	0.1	0.1	RI, MS
7	Nonan-2-one	1077	tr	0.1	0.1	RI, MS
8	Nonanal	1081	0.4	0.2	0.3	RI, MS
9	(E)-2-nonenal	1133	0.4	0.3	0.2	RI, MS
10	(Z)-2-nonen-1-ol	1155	0.1	0.4	0.4	RI, MS
11	Octanoic acid	1174	0.1	tr	tr	RI, MS
12	1-decen-3-ol	1181	0.6	tr	tr	RI, MS
13	Decanal	1183	tr	0.2	0.2	RI, MS
14	3-dodecen-1-yne	1214	0.1	tr	tr	RI, MS
15	Carvone	1225	tr	tr	tr	RI, MS
16	(E)-2-decanal	1251	tr	0.2	0.2	RI, MS
17	(E,E)-2,4-decadienal	1289	0.3	0.3	0.3	RI, MS
18	β-ionone	1454	0.2	0.2	0.2	RI, MS
19	3,4-dimethyl-5-pentyl-5H-furan-2-one	1486	1.9	1.0	1.0	RI, MS, Ref
20	γ-undecalactone	1524	tr	tr	0.2	RI, MS, Ref
21	Dodecanoic acid	1547	0.1	tr	tr	RI, MS
22	Hexadecanoic acid	1968	1.4	0.8	0.8	RI, MS
23	falcarinol	2026	86.9	93.1	90.6	RI, MS
24	α-kaurene	2049	0.1	0.3	0.2	RI, MS
Total identification (%)			95.1	98.3	96.2	
EO yields (%) (w/w)			0.05	0.04	0.03	
Oxygenated compounds			92.8	95.4	92.7	
Hydrogenated compounds			2.3	2.9	3.5	

^a^Order of elution is given on apolar column (SPB-1)

^b^Retention indices on SPB-1 column (RI_a_)

^c^Essential oils of *E. triquetrum* stems. Quantification was carried out using RFs relative to tridecane as internal standard

*Abbreviations*: %, normalized percentages are given on the apolar column; tr, trace (< 0.05%); RI: retention index; MS: mass spectrometry in electronic impact mode. All compounds were identified by comparing their EI-MS and retention indices with references compiled in the in-house library except for compounds 19 and 20; EO: essential oil. Yield is based on the fresh weight of the stems; Ref, Compounds identified with references from literature data and compiled in the in-house library

### Toxic activity of essential oil from *Eryngium triquetrum* against adult *B. glabrata* snails

**Fig. 1:**
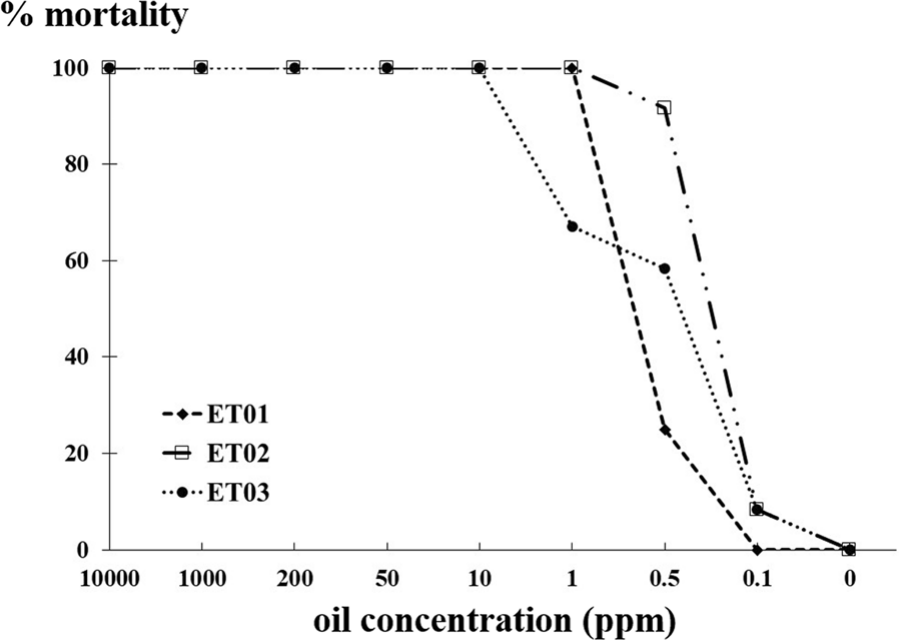
Mortality rates of *Biomphalaria glabrata* at different concentrations of essential oil extracted from *Eryngium triquetrum*. Three independent fractions of essential oil extracts were tested and noted ET01, ET02 and ET03. Their respective compositions are reported in Table 2 Mortality rate was obtained by exposing 12 snails for each different concentrations of each essential oil

We independently assessed trial toxicity of three extractions of essential oil from *E. triquetrum* against adult *B. glabrata*, the vector snail of the trematode *S. mansoni.* At concentrations above 10 ppm, the mortality rate reached 100% whereby most of the exposed snails retracted into their shells with hemolymph expulsion. From concentrations of 0.5 to 1 ppm, the survival rate of exposed snails varied slightly between the three independent batches of oil extracted. Based on Probit analysis using the survival of treated snails to *E. triquetrum* oils, the concentration required to produce 50% mortality for deaths to 24 h after exposure (LC_50_) was 0.51, 0.63, 0.69 ppm for ET02, ET03 and ET01 respectively (Fig. 1). As the different LC_50_ values were relatively close, the following toxicity assays were performed with ET03 essential oil extract. Their LC_90_ values were 0.9, 1.07 and 1.08 respectively.

### Toxic activity of *Eryngium triquetrum* extract against infected snails

**Fig. 2:**
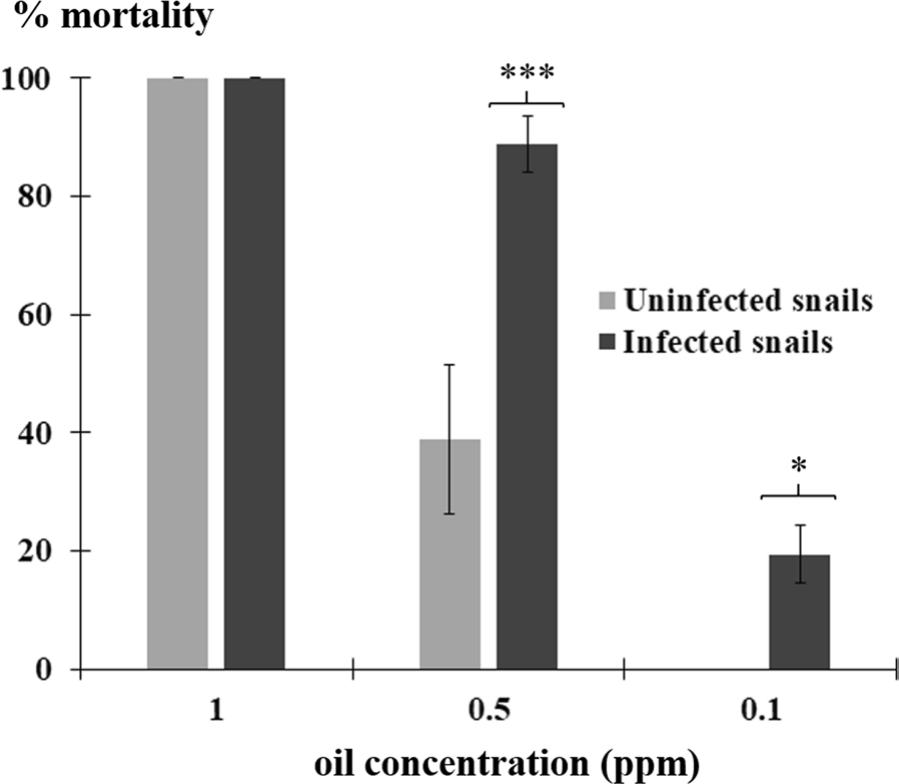
Effect of the essential oil extracted from *Eryngium triquetrum* in snails infected with *Schistosoma mansoni*. Results from 3 independent experiments are presented as the mean ± standard deviation (SD); 20–30 snails were submitted to different doses of essential oil. Statistical significance was determined through Fisherʼs exact test (*P* = 0.0008 and 0.0496 for comparisons at 0.5 and 0.1 ppm, respectively, between infected and uninfected snails). Asterisks indicate significant differences between uninfected and infected snails

The molluscicidal activity of the essential oil from *E. triquetrum* was also tested against snails infected with *S. mansoni*. Different essential oil concentrations around the LC_50_ were used under the same conditions as those described previously. Interestingly, this plant-derived molluscicide seems to be more potent against infected snails. Indeed, no toxic effects were observed in non-infected snails for a concentration of 0.1 ppm while about 20% of infected snails were killed after 24 h exposure. At 0.5 ppm, the essential oil of *E. triquetrum* was 2 times more toxic to parasitized snails with a mortality rate of 88.8 ± 4.8% whereas it was 38.9 ± 12% in the non-infected snails (Fig. 2).

### Embryotoxicity evaluation on snail eggs

**Fig. 3:**
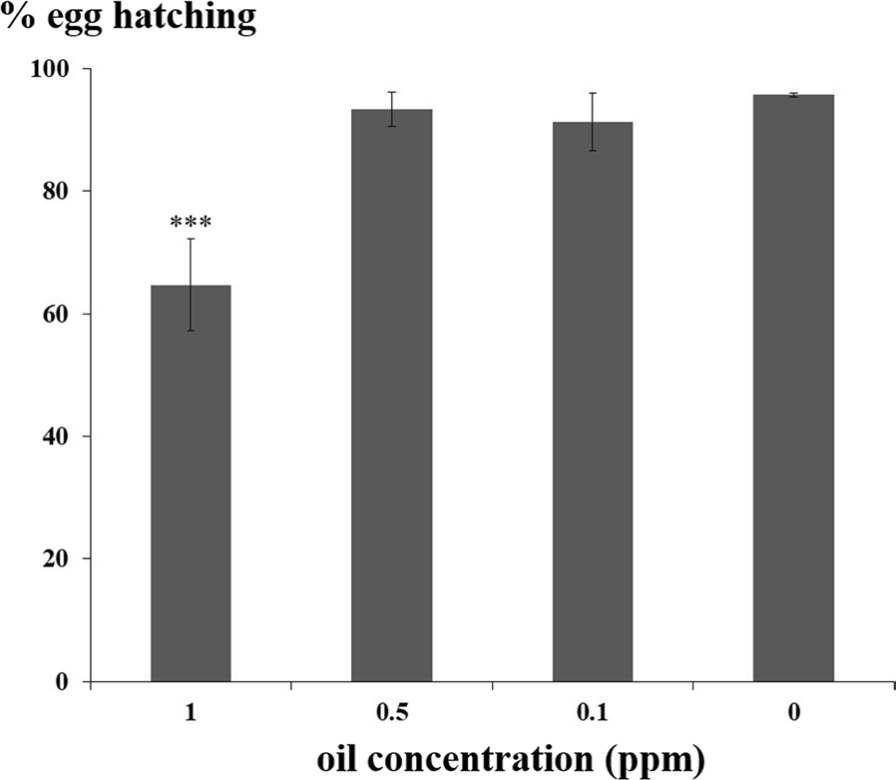
*Biomphalaria glabrata* eggs and embryos exposed to essential oil from *Eryngium triquetrum.* The data are presented as the mean ± standard deviation (SD). After 24 h of exposure to different concentrations of essential oil, egg hatching and snail development was monitored during 20 days. The term egg hatching corresponds here to a hatching leading to a normal development of the snail. This toxicity test was carried out twice on more than 25 egg masses per condition. Statistical significance was determined through Fisher exact test (*P* < 0.001). Asterisks indicate a significant difference between treated and non-treated eggs

*Eryngium triquetrum* extract was toxic to adult snails (infected or uninfected) in a dose-dependent manner (Figs. 1, 2). For the same range of concentrations, it appears that the egg masses are less sensitive to the essential oil extract. No difference of toxicity and lethality was observed in eggs exposed to concentrations below and around the LC_50_. The positive hatching rates were 91.3 ± 4.7%, 93.3 ± 2.8% at the concentrations of 0.1 and 0.5 ppm, respectively compared to the control which yielded 95.7± 0.3% hatching. Embryos and adult *B. glabrata* snails showed different sensitivity to the plant extract since the lethal dose for 100% of the adult snail population (1 ppm) showed moderate embryonic lethal effect, 64.7 ± 7.5% of snail embryos hatched and developed successfully (Fig. 3).

### Parasiticidal activity against the free-swimming larval stages of *S. mansoni* and effect on snail-parasite compatibility

**Fig. 4:**
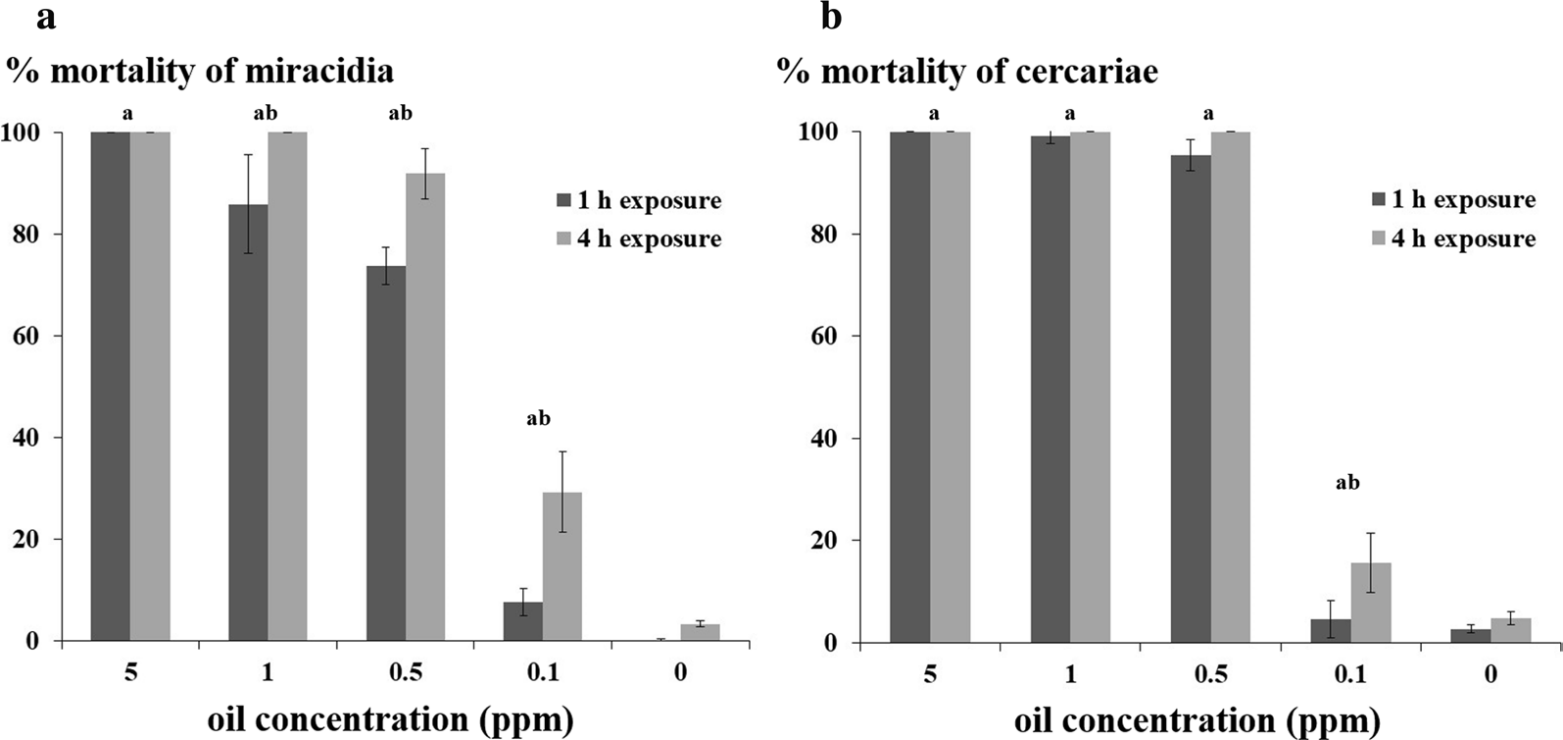
Mortality of free-swimming larvae of *Schistosoma mansoni* exposed to essential oil from *Eryngium triquetrum*. Results are presented as the mean ± standard deviation (SD) for at least 3 and 2 independent replicates for toxicity assays against miracidia and cercariae, respectively. About 300 miracidia and 100 cercariae were used for each independent experiment. The parasites (miracidia (**a**) and cercariae (**b**)) are considered dead if not mobile or motionless at the bottom of plates. Statistical significance was determined through Fisher exact test; “a” indicates a significant difference between the treated sample and the control, “b” indicates a significant difference between 1-h treated and 4-h treated parasites

**Fig. 5:**
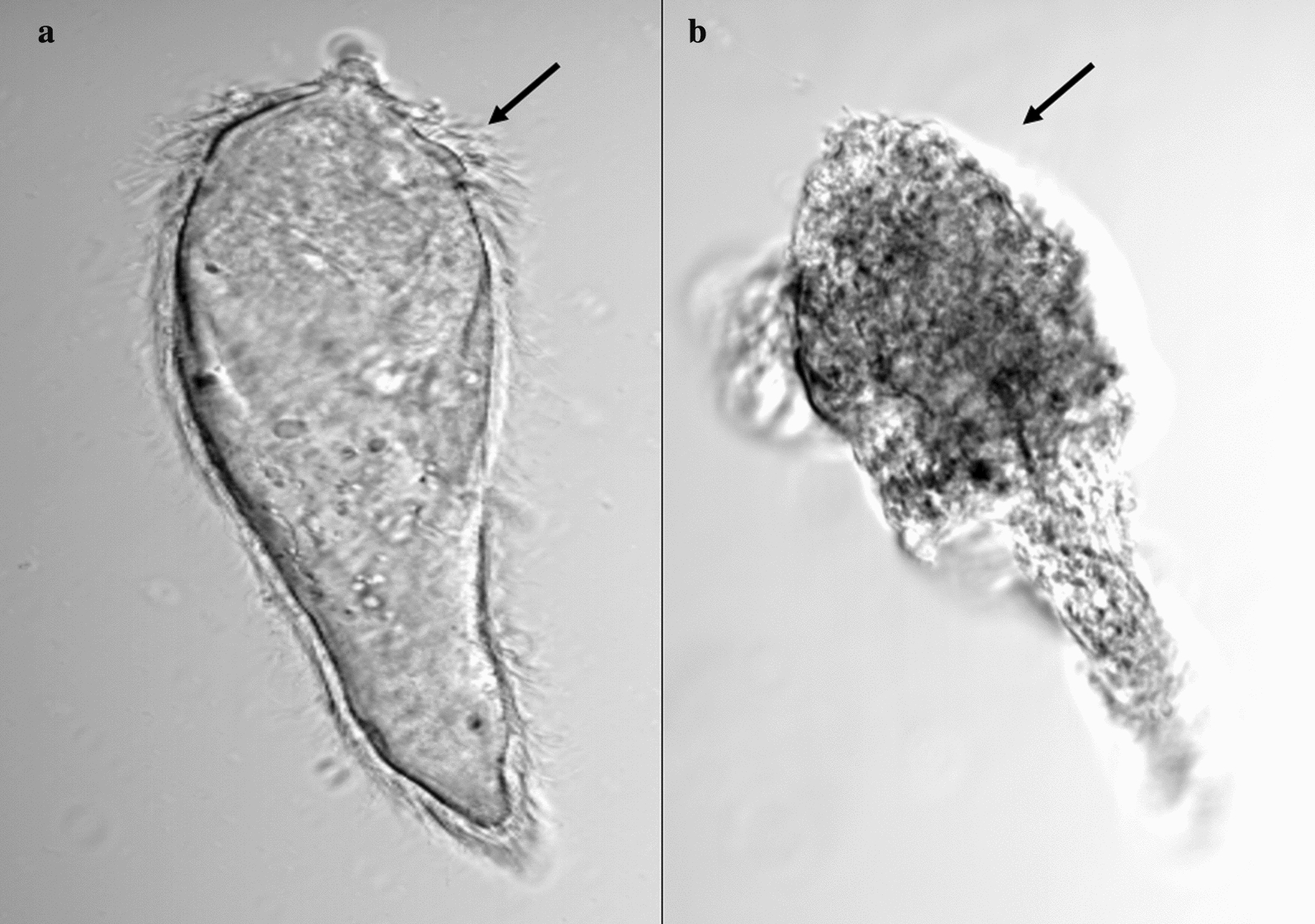
Observation of parasiticidal activity on miracidia. **a** Miracidium not exposed to *E. triquetrum* essential oil. **b** Miracidum exposed to 0.5 ppm during 1 h. Note surface alterations on miracidium exposed to *E. triquetrum* extract with total loss of ciliated plates (arrows)

**Fig. 6:**
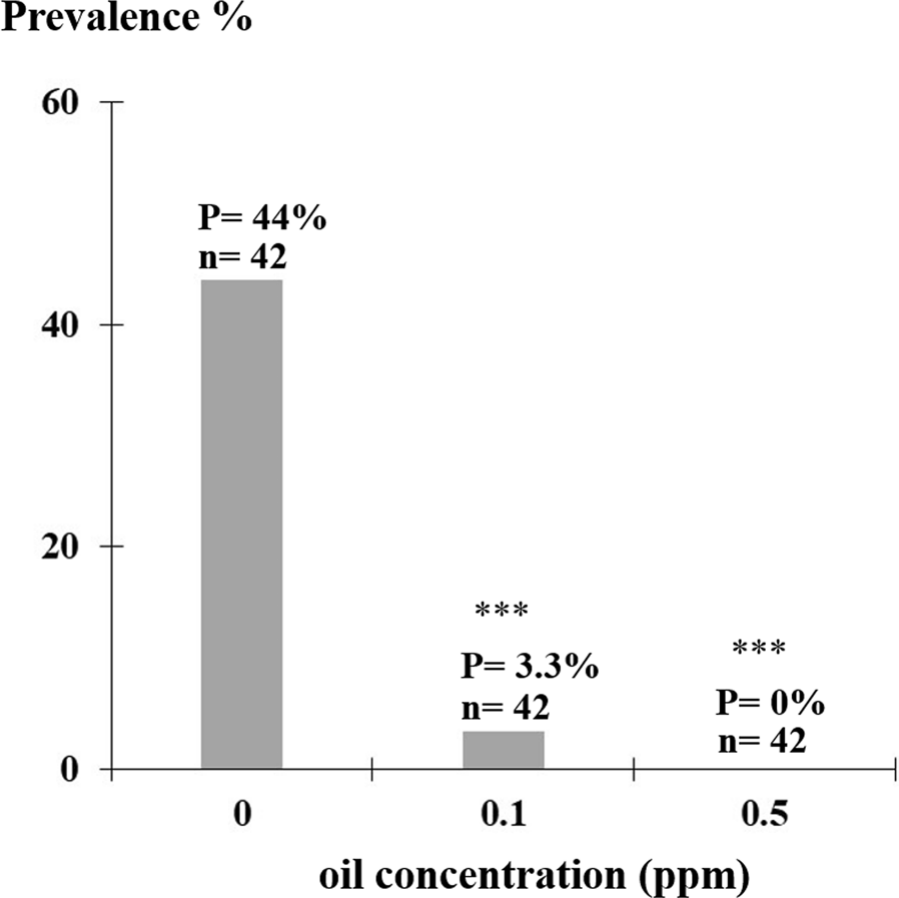
Effect of *Eryngium triquetrum* extract on *Schistosoma mansoni* miracidia infectivity. Prevalence of snails exposed to miracidia treated with essential oil was assessed *via* cercarial shedding; 42 snails were individually exposed to one miracidium exposed to 0.1 or 0.5 ppm of plant extract during a short exposure (30 min). All miracidia recovered were alive and swam toward light source. Data are presented as proportions of infected snails ± standard error (*n* = 42). Statistical significance was determined through Fisherʼs exact test (*P* < 0.00001). Asterisks indicate a significant difference between treated and non-treated miracidia

An alternative approach in control of schistosomiasis instead of controlling the snail population could be to focus on the free-swimming larval stages of the parasite, miracidia and cercariae. These parasite larval stages were exposed for 1 or 4 h to different concentrations of *E. triquetrum* extract ranging from 0.1 to 1 ppm to evaluate its potential lethal effect. The mortality of the miracidia and cercariae of *S. mansoni* was nearly complete for 5, 1 and 0.5 ppm of oil extract irrespective of the exposure time. For a concentration of 0.1 ppm, the mortality rate of miracidia dropped considerably reaching 7.6 ± 2.7% and 29.3 ± 8.0% with 1 and 4 hours of exposure (Fig. 4a). After a relatively short exposure time, severe surface damage on miracidia can be observed with a general loss of cilia that probably cause their immobility (Fig. 5). The mortality pattern for exposed cercariae was quite similar with 4.6 ± 3.6% and 15.6 ± 5.8% of deaths observed after the different contact times (Fig. 4b). To determine the ability of exposed miracidia to infect their host, sublethal concentrations of the essential oil from *E. triquetrum* were applied for 30 min. Results of the snail exposure to treated *S. mansoni* miracidia are shown in Fig. 6. Miracidia exposed to low concentrations of plant extract were less infective with a prevalence of 3.3% compared to untreated miracidia (prevalence of 44%).

## Discussion

The eradication of schistosomiasis provided by the WHO’s road map and planned for 2030 requires a significant investment from all disciplinary fields to develop new drugs against human schistosomes and to interrupt the parasite life-cycle. Even if the study of biological interactions between *Schistosoma* and its snail vector has taken a new dimension based on their genome sequencing studies [34, 35], the CRISPR production of parasite-resistant transgenic snails or genetically modified sterile worms are quite far from having any results because of technical difficulties and little-known ecological aspects of such releases [36, 37]. Currently, massive treatment with praziquantel and improving sanitation appear the best way to control the disease. However, new foci are appearing, and other are re-emerging probably due to global warming [38, 39] and mass human migration [40], but also to the emergence of drug-tolerant schistosomes [41, 42]. In this context, snail control appears to be a crucial strategy to ensure a sustainable control and elimination of schistosomiasis. A number of molluscicides have been developed in an attempt to decrease parasitic disease transmission. Among synthetic molluscicides, niclosamide is the most widely employed in official schistosomiasis control programmes [43, 44] although concerns about the emergence of resistant snail population remains [12] and its apparent toxicity to other living organisms has been observed [11, 45, 46]. In addition, it is often necessary to repeat spraying in treated transmission sites due to snail repopulation [44]. Consequently, increasing efforts focused on identifying and characterizing new plant-derived molluscicides is required as a safer alternative [43, 44]. Among the molluscicidal agents extracted from plants, the latex from *Euphorbia* sp. appears promising by acting on both the snail vector and the parasite [13]. The LC_50_ can depend on several parameters such as *Euphorbia* species, the different part of the plant or the solvent used for latex extraction [47]. For example, LC_50_ of latex extracted from *E. umbellata* range from 1.36 to 40 ppm [45, 48] and around 30 ppm and 0.5 from *E. tirucalli* and *E. splendens*, respectively [49, 50]. Others plant extracts also display molluscicidal activities at concentrations lower that the one required by the WHO. Alcoholic stem extract from *Jatropha gossypiifolia* shows toxicity towards adult snails after 24 h exposure with a LC_50_ of 25 ppm [51]. Chlorophyllin extracted from *Moringa oleifera* leaves has a lethal activity on adults *Biomphalaria* snails exposed during 24 h probably by interfering with nervous and digestive systems [52]. A *Camellia oleifera* extract enriched in saponin exhibits a high level of toxicity against adult *B. alexandrina* snails for which LC_50_ is quite similar to niclosamide, used as reference [14]. Other plant-derived terpenes such as jatrophone have also a high toxicity against adult snails [53, 54]. This list of plant-derived extracts with a potential molluscicidal activity is far from being exhaustive. Moreover, a recent review updates the different essential oils used for their toxic effect against schistosomiasis transmitting snails [55]. The most studied plants are those belonging to families Lamiacae, Rutaceae and Myrtaceae. The major chemical components determined by chromatography analyses are the terpenic derivatives for which their concentration in oils depend strongly on the nature of organic solvent and the different parts of plants used [55, 56]. To our knowledge, the present study is the first to report the molluscicidal and parasiticidal activities of an essential oil extracted from an Apiaciae, *Eryngium triquetrum*. Its biological activities can be assigned to the high falcarinol content (about 90%), an aliphatic polyacetylene molecule produced by several plants such as carrots [57]. Interestingly, the high level of this component is only found in *E. triquetrum* and could be considered as a main source of production. Indeed, essential oils of *Eryngium* species such as *E. campestre*, *E. maritimum* or *E. alpinum* do not contain such falcarinol compound or at lower concentrations [58–60]. As for *E. planum*, the maximal rate of falcarinol (about 60%) is concentrated in roots which make it difficult to benefit from its biological activity [61]. As *E. triquetrum* from Algeria is a falcarinol-rich essential oil, especially from stems, exploitation at large farming scale could be proposed for molluscicidal but also therapeutic application. Indeed, falcarinol and/or essential oil from *E. triquetrum* display antimicrobial effect as well as anti-inflammatory properties [23, 59]. Also, falcarinol could be used in tumor treatment since cytotoxic activity against human cancer cell lines has been demonstrated [59, 62].

In the present study, the essential oil from *E. triquetrum* seems to be a promising molluscicidal candidate considering the standard criteria established by the WHO with a lethal concentration of 90% and 50% below 400 and 100 ppm respectively. The LC_90_ and LC_50_ values against adult *B. glabrata* snails of 1.02 and 0.61 ppm, respectively, are significantly lower than most of the well-characterized essential oils and similar to latex or niclosamide. Unlike the reference molluscicide, the lower toxicity of the essential oil from *E. triquetrum* to embryonated eggs potentially due to a low penetrating ability into egg masses, suggests a higher specificity of action towards adult snails. This toxic dichotomy can be useful to treat the aquatic environment while reducing the side effects on non-target animals. The disruption of parasite’s life-cycle is more favored when snails are even early infected. This higher mortality in infected snails could be linked to a broad dysregulation of signaling pathways involved in detoxification [63] and/or energy production metabolism [13, 64].

In a context of schistosomiasis elimination goal, the characterization of biological activities from plant-derived compounds mainly focuses on molluscicidal toxicity to break the parasite transmission cycle. However, some promising candidates present a dual activity by causing either the death of infective larvae [16, 17, 65–67] or developmental abnormalities leading to a low pathogenicity [68]. Mortality values after being exposed only for 24 h at 0.5 ppm were around 75% and 95% for miracidia and cercarciae, respectively, and close to 100% for both after 4 h exposure. Interestingly, no snail death was observed under the same experimental conditions (Additional file 1: Figure S1). This falcarinol-enriched extract from *E. triquetrum* also prevents parasite infestation. Indeed, at low level concentrations during a short time exposure (30 min), the snail-parasite compatibility is strongly affected with no parasitized snails at 0.5 ppm. This significant loss of parasite virulence following treatment could be attributed to the shedding of the ciliated plates used by parasites for locomotion and penetration into the snail host (Fig. 5).

## Conclusions

We present a complete analysis of biological activities of a falcarinol-enriched extract from an Apiaceae ranging from molluscicidal and parasiticidal activities to the infection capacity of the exposed parasites. The extract from *E. triquetrum* has shown promising results as an alternative molluscicidal and parasiticidal environmentally compatible agent to reduce snail infections with schistosomes in transmission foci. Future research could investigate the biological activity of synthetic or natural falcarinol and its derivatives to develop new agents to prevent schistosomiasis transmission.

## Supplementary information

**Additional file 1: Figure S1.** Effect of short-time exposure to the essential oil extracted from *Eryngium triquetrum* on uninfected snails. Data are summarised as the mean and standard deviation (SD). Statistical significance was determined through Fisherʼs exact test. *P*-value < 0.05 was considered statistically significant. A “a” indicates a significant difference between treated sample and control (Fischer’s exact test; *P* < 0.001); and “b” indicates a significant difference between 1 h treated and 4 h treated snails (Fisher’s exact test; *P* < 0.001).

## Data Availability

The datasets used during the present study are available from the corresponding author on reasonable request.

## Abbreviations

ET: *Eryngium triquetrum*
LC: lethal concentration
MS: mass spectrometry
RI: retention index
WHO: world health organization
