# Supplementary figures and images for: Molluscicidal and parasiticidal activities of Eryngium triquetrum essential oil on Schistosoma mansoni and its intermediate snail host Biomphalaria glabrata, a double impact
Source: Parasit Vectors. 2020 Sep 23;13:486. doi: 10.1186/s13071-020-04367-w (PMC7513307; doi:10.1186/s13071-020-04367-w)

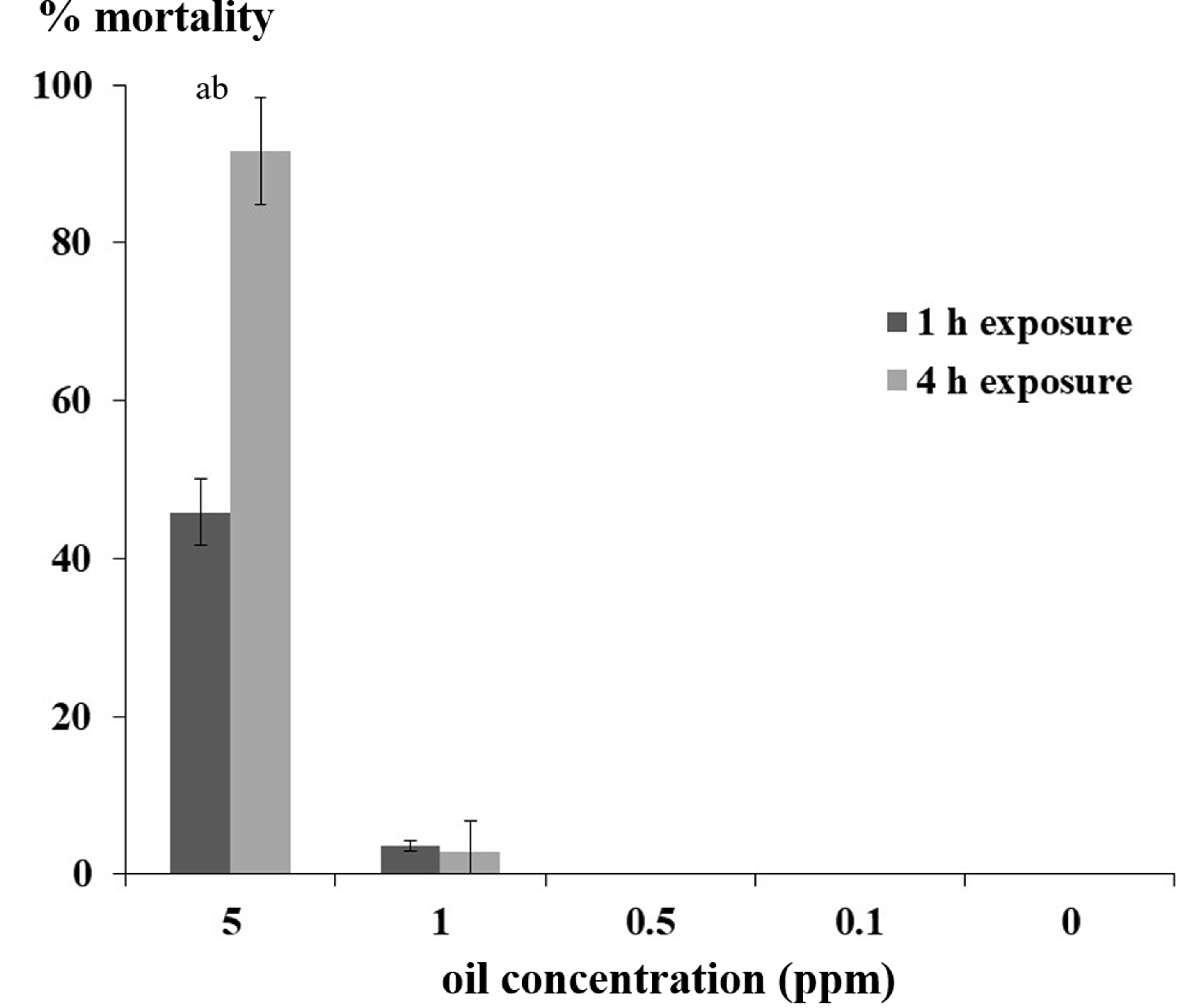

Supplement: Supplementary file 1 — Additional file 1: Figure S1. Effect of short-time exposure to the essential oil extracted from Eryngium triquetrum on uninfected snails. Data are summarised as the mean and standard deviation (SD). Statistical significance was determined through Fisherʼs exact test. P-value < 0.05 was considered statistically significant. A “a” indicates a significant difference between treated sample and control (Fischer’s exact test; P < 0.001); and “b” indicates a significant difference between 1 h treated and 4 h treated snails (Fisher’s exact test; P < 0.001). [file 13071_2020_4367_MOESM1_ESM.tif]
